# Transparency and stability of low density stellar plasma related to Boltzmann statistics and to thermal bremsstrahlung

**DOI:** 10.1038/s41598-019-56784-2

**Published:** 2019-12-31

**Authors:** Y. Ben-Aryeh

**Affiliations:** Technion-Israel Institute of Technology, Physic Department, Haifa, 32000 Israel

**Keywords:** Astronomy and planetary science, Optics and photonics, Physics

## Abstract

The stability of low density stellar plasma is analyzed for a star with a spherical symmetry which is in equilibrium between the gravitational attractive forces and the repulsive pressure forces of an ideal electron gas where the analysis is developed by the use of Boltzmann statistics. Fundamental results are obtained for the radius and total mass of such star and its gravitational forces are large due to the extreme large volume. The absorption and emission of radiation for extremely low density star plasmas is very small over the entire electro-magnetic spectrum.

## Introduction

In the present work we are interested in the analysis of low density ionized stellar plasma with relatively high temperature for which the Boltzmann statistics is valid. We assume that the plasma behaves as a perfect gas characterized by the fact that the average interaction of the particles is smaller than the thermal energy^1^. For a perfect gas i.e., when the plasma is sufficiently rarefied, with electron density *n*_*e*_ (*cm*^−3^) at temperature *T*, the electrons pressure *P*_*e*_ is given by

1 $${P}_{e}={n}_{e}{k}_{B}T,$$

where *k*_*B*_ is Boltzmann constant, *T* the absolute temperature and *n*_*e*_ is the number of ionized-electrons per unit volume.

Completely ionized plasma behaves as a perfect gas at low densities. In fact, plasma which is partly ionized behaves as a perfect gas at densities lower than a critical value. This critical value can be found by comparing the Coulomb interaction energy ${E}_{C}\simeq {e}^{2}/\bar{r}$ (where $e\simeq 4.8\cdot {10}^{-10}{g}^{1/2}c{m}^{3/2}{s}^{-1}$ is the electron charge in CGS units and $\bar{r}$ is the average separation of the particles) with the thermal energy ${E}_{T}=(3/2){k}_{B}T$. Equation (1) is valid under the condition ${E}_{C}\ll {E}_{T}$. In the present work we study the stability of a star under perfect gas conditions where the star stability is produced by the balance between the gravitational forces and the pressure produced by perfect gas conditions, and find the impact of such model on the properties of low density plasmas. While the star stability was studied under high electron densities using Fermi-Dirac statistics^2–6^ we study here the problem for low densities with relatively high temperature using Boltzmann statistics.

Quantum effects for low density ionized stellar plasmas, with one ionic component, with atomic number *Z* (satisfying Boltzmann statistics) can be neglected under the condition $Z{e}^{2}/\hslash v\gg 1$, where $\hslash$ is Planck constant (divided by $2\pi$) and *v* the averaged electron velocity^7–10^. Similar relations can be obtained if we have more than one ionic component. Under the condition: $Z{e}^{2}/\hslash v\ge 1$ we can still use a classical analysis and the quantum effects result as corrections to the classical formulas (Gaunt factors^11^ which under this condition are small numbers). For Boltzmann statistics we use the approximation $v=\sqrt{\frac{3{k}_{B}T}{{m}_{e}}}$ where *m*_*e*_ is the electron mass and then this relation can be inserted in the condition $Z{e}^{2}/\hslash v\gg 1$.

We should take into account that the present use of Boltzmann statistics for the average velocity of the electrons and the radiation pressure of Eq. (1) are only approximate values which are used for estimating the order of magnitudes for the radius and mass of the low density star. In a more accurate description one needs to take into account the thermal escape of particles (which are in the high velocity region of the Boltzmann distribution) from the low density star. Such effect is similar to that obtained in evaporative cooling and has been treated by using a truncated Boltzmann distribution^12^. In the present work I do not consider this effect but I hope to study it in future work.

In the present work we treat thermal bremsstrahlung effects influencing the absorption and and emission of electromagnetic radiation by low density plasma. We find that such radiative processes are proportional to the products of the electrons and ions densities so that they become very small for very low density plasmas.

For simplicity of discussion we treat a star with spherical symmetry where its stability is related to gravitational forces without any other external perturbations e.g. magnetic fields etc. Magnetic fields might be important for high density stars^13^. But as shown quite long ago by Van Vleck^14^, following Bohr-Van-Leeuven theorem, when classical mechanics, including Boltzmann statistics and free electrons models are applied, the thermal averaged value of the magnetic fields vanishes. Such local classical kinetic equilibrium (LCKE)^15^ is valid under the conditions: 1) Quantum effects are negligible in the plasma. 2) None of the distribution functions are time dependent. 3) The distribution function is Maxwellian. As such conditions are assumed in the present analysis we neglect magnetic fields effects. The stability conditions derived in the present work, for low density plasma, imply that as the plasma density is decreased the star radius is increased. As the gravitational forces are proportional to the star volume i.e. to *R*^3^, where *R* is the star radius, gravitational forces are significant also for low density plasmas.

Dark matter is thought in previous works to be non-baryonic, possibly being composed of some as-yet undiscovered particles^16–18^. There is not any evidence to the existence of such non-baryonic matter and its nature referred in the literature is obscure. In the preset work I discuss the possibility that the present analysis describes dark matter. Usually stars are observed either by radiation emitted by them or by reflected and transmittance of light incident on them from other sources. It is interesting to note by following the present analysis that stellar plasmas with very low densities do not emit any significant radiation and their transmittance is nearly equal 1 (reflectance nearly zero) so that they have the dark matter properties but with baryonic components. By following the conditions for stability of low density star plasma developed in the present work we find that for such star with spherical symmetry, strong gravitational effects occur due to the extremely large volume of such star. Therefore the existence of strong gravitational forces does not imply that such matter is non-baryonic especially as there is not yet any experimental evidence that such non-baryonic matter exists. Later in the discussion I describe certain properties of dark matter related to the present analysis and compare the present analysis with some other works describing dark matter.

## The Stability of Stellar Plasmas With Low Densities Under Boltzmann Statistics

For simplicity of discussion we treat a star with spherical symmetry where its stability is related to gravitational forces without any other external perturbations. We simplify the treatment by assuming one ionic component with atomic number *Z* but the analysis can easily be generalized if we have more ionic components.

The gravitational force *g* at a distance *r* from the star center is due entirely to the mass *M*_*r*_ interior to this distance:

2 $$g=-\,G{M}_{r}/{r}^{2},$$

where *G* is the constant of gravitation. Assuming that $\phi$ is the gravitational potential then

3 $$g=-\,d\phi /dr.$$

According to the hydrostatic equation

4 $$dP=g\rho dr$$

where *P* is the star plasma pressure and $\rho$ the star mass density, both are functions of distance *r* from the star center. Inserting Eq. (3) into Eq. (4), we get:

5 $$dP=-\,\rho d\phi $$

This equation describes the decrease of the plasma pressure as we move to larger values of *r* balancing the attractive gravitational forces.

Assuming that the star plasma behaves as an ideal gas then the pressure *P* is given by

6 $$P={n}_{e}{k}_{B}T$$

Assuming also that the gradient of temperature is small relative to the gradient of the electron density *n*_*e*_ (isothermal process) then we get:

7 $$dP={k}_{B}Td{n}_{e}=-\,\rho d\phi $$

If there are $\kappa$ nucleons for each ionized electron, then the mass density is given approximately by

8 $$\rho =\kappa {n}_{e}{m}_{N}.$$

here ${m}_{N}\approx 1.67\cdot {10}^{-24}g$ is the mass of the nucleon and ${n}_{e}(c{m}^{-3})$ is the density of ionized electrons. By inserting Eq. (8) into Eq. (7) and assuming that at the star center the potential $\phi$ vanishes and its ionized electron density is *n*_0_ we get:

9 $$\begin{array}{c}\frac{d{n}_{e}}{d\phi }=-\,\frac{\kappa {m}_{N}}{{k}_{B}T}{n}_{e}\to \,\mathrm{ln}({n}_{e})+C=-\,\frac{\kappa {m}_{N}}{{k}_{B}T}\phi ,\,C=-\,\mathrm{ln}({n}_{0})\to \,\mathrm{ln}(\frac{{n}_{e}}{{n}_{0}})=-\,\frac{\kappa {m}_{N}}{{k}_{B}T}\phi \\ {n}_{e}(r)={n}_{0}\,\exp \,[-(\frac{\kappa {m}_{N}}{{k}_{B}T})\phi (r)];\,\phi (r=0)=0;\,{n}_{e}(r=0)={n}_{0}\end{array}$$

This equation describes the decrease of the ionized electrons density ${n}_{e}(r)$ as a function of the exponential of $\phi (r)$. Here $\phi (r)$ is changing from zero to large values, as function of the distance *r* from the star center. Our aim in the following analysis is to find the change of ${n}_{e}(r)$ as function of the distance *r* from the star center. In these calculations we assume that *n*_0_ is the density of ionized electrons in the star center which is taken as an experimental parameter. In order to find the dependence of the electron density ${n}_{e}(r)$ on the distance *r* from the star center we need to take into account the Poisson’s equation for the potential $\phi$ which for a star with spherical symmetry has the form:

10 $$\frac{{d}^{2}\phi (r)}{d{r}^{2}}+\frac{2}{r}\frac{d\phi (r)}{dr}=4\pi G\rho (r)$$

here $\rho (r)$ is proportional to the ionized electron density ${n}_{e}(r)$, as given by Eq. (8), and *G* is the gravitational constant. Substituting the relation $\rho =\kappa {n}_{e}{m}_{N}$, from Eq. (8) into Eq. (10), and by using relation (9) for ${n}_{e}(r)$ we get

11 $$\frac{{d}^{2}\phi (r)}{d{r}^{2}}+\frac{2}{r}\frac{d\phi (r)}{dr}=4\pi G\kappa {m}_{N}{n}_{e}(r)=4\pi G\kappa {m}_{N}{n}_{0}\,\exp \,[-(\frac{\kappa {m}_{N}}{{k}_{B}T})\phi (r)]$$

We note that on the right side of Eq. (11) appears an exponential function of the potential $\phi (r)$ with a very small coefficient given by $-\frac{\kappa {m}_{N}}{{k}_{B}T}$. One should notice that the coefficient before the exponential is also a very small number for low electron densities. It is difficult to get analytical explicit solutions to this equation, as series expansion of this exponential function converges very slowly for large values of *r*. In the following analysis we transform Eq. (11) to differential equation of ${n}_{e}(r)$. It will give after some calculations the change of ${n}_{e}(r)$, from its initial value ${n}_{e}(r=0)={n}_{0}$ at the star center (taken as an experimental parameter) to smaller values as a function of the distance *r* from the star center.

According to Eq. (9) we get

12 $$\begin{array}{c}\frac{\partial {n}_{e}(r)}{\partial r}=-\,(\frac{\kappa {m}_{N}}{{k}_{B}T})\frac{\partial \phi (r)}{\partial r}{n}_{e}(r)\to \frac{\partial \phi (r)}{\partial r}=-\,\frac{1}{{n}_{e}(r)}\frac{\partial {n}_{e}(r)}{\partial r}(\frac{{k}_{B}T}{\kappa {m}_{N}})\\ \frac{{\partial }^{2}\phi (r)}{\partial {r}^{2}}=[\frac{1}{{n}_{e}{(r)}^{2}}{(\frac{\partial {n}_{e}(r)}{\partial r})}^{2}-\frac{1}{{n}_{e}(r)}\frac{{\partial }^{2}{n}_{e}(r)}{\partial {r}^{2}}](\frac{{k}_{B}T}{\kappa {m}_{N}}).\end{array}$$

Inserting Eq. (8) and the potential derivatives according to Eq. (12) into Eq. (10) we get

13 $$[\frac{1}{{n}_{e}{(r)}^{2}}{(\frac{{\rm{\partial }}{n}_{e}(r)}{{\rm{\partial }}r})}^{2}-\frac{1}{{n}_{e}(r)}\frac{{{\rm{\partial }}}^{2}{n}_{e}(r)}{{\rm{\partial }}{r}^{2}}-\frac{1}{{n}_{e}(r)}\frac{2}{r}\frac{{\rm{\partial }}{n}_{e}(r)}{{\rm{\partial }}r}](\frac{{k}_{B}T}{\kappa {m}_{N}})=4\pi G\kappa {m}_{N}{n}_{e}(r).$$

Multiplying Eq. (13) by ${n}_{e}(r)$ and rearranging its terms we get:

14 $$-\frac{{{\rm{\partial }}}^{2}{n}_{e}(r)}{{\rm{\partial }}{r}^{2}}-\frac{2}{r}\frac{{\rm{\partial }}{n}_{e}(r)}{{\rm{\partial }}r}+\frac{1}{{n}_{e}(r)}{(\frac{{\rm{\partial }}{n}_{e}(r)}{{\rm{\partial }}r})}^{2}=4\pi G\frac{{(\kappa {m}_{N})}^{2}}{{k}_{B}T}{n}_{e}{(r)}^{2}$$

We define $\theta (r)=\frac{{n}_{e}(r)}{{n}_{0}}$, and divide Eq. (14) by ${n}_{0}$ then we get

15 $$\begin{array}{c}-\frac{{{\rm{\partial }}}^{2}\theta (r)}{{\rm{\partial }}{r}^{2}}-\frac{2}{r}\frac{({\rm{\partial }}\theta (r))}{{\rm{\partial }}r}+\frac{1}{\theta (r)}{(\frac{{\rm{\partial }}\theta (r)}{{\rm{\partial }}r})}^{2}=4\pi G\frac{{(\kappa {m}_{N})}^{2}{n}_{0}}{{k}_{B}T}\theta {(r)}^{2},\\ \theta (r)=\frac{{n}_{e}(r)}{{n}_{0}};\,{n}_{0}={n}_{e}(r=0)\end{array}$$

We define

16 $$\xi =4\pi G\frac{{(\kappa {m}_{N})}^{2}{n}_{0}}{{k}_{B}T}=1.69\cdot {10}^{-38}\frac{{n}_{0}{\kappa }^{2}}{T}(c{m}^{-2});\,x=r\sqrt{\xi }\simeq r\cdot 1.3\cdot {10}^{-19}\kappa \sqrt{\frac{{n}_{0}}{T}}$$

We divide Eq. (15), for $\theta (r)$ by $\xi$. Then, this equation can be written as:

17 $$-\frac{{{\rm{\partial }}}^{2}\theta (x)}{{\rm{\partial }}{x}^{2}}-\frac{2}{x}\frac{({\rm{\partial }}\theta (x))}{{\rm{\partial }}x}+\frac{1}{\theta {(x)}^{2}}{(\frac{{\rm{\partial }}\theta (x)}{{\rm{\partial }}x})}^{2}=\theta {(x)}^{2}$$

Equation (17) describes the change of the normalized electron density ${n}_{e}(x)/{n}_{0}=\theta (x)$ as function of the normalized distance $x=r\sqrt{\xi }$ from the star center. This equation describes also the change of the mass density $\rho (g\cdot c{m}^{-3})=\kappa {n}_{e}{m}_{N}$ $({m}_{N}=1.67\cdot {10}^{-24}g)$ as function of the distance from the star center. Here ${m}_{N}$ is the mass of nucleon and $\kappa$ is the ratio between the density of nucleons and that of electrons (e.g. for completely ionized Hydrogen plasma $\kappa =2$). By neglecting the third term on the left side of Eq. (17) we obtain a differential equation which was solved numerically and was given as the Lane-Emden equation with $n=2$^19^. It shows the change of the value of $\theta (x)=1$ for $x=0$ at $r=0$, to the value $\theta (x)=0$ for $x=1=\sqrt{\xi }\,R$ where $R=1/\sqrt{\xi }$ is approximately the star radius. Such behavior occurs also for the solutions of the full Eq. (17), but since the third term, on the left side of Eq. (17), cannot be neglected the form of change of $\theta (x)$ is different from that of Lane-Emden equation. Like the Lane-Emden equation the range $x=1$ describes approximately the range of values of $\theta (x)$, and substituting in Eq. (16) the value $x=1=R\sqrt{\xi }$ (where $\xi$ in the present conditions is extremely small number) we find that the radius *R* of the star stretches over a very long distance which is of order

18 $$R(star\,radius)\simeq \frac{1}{\sqrt{\xi }}\simeq \sqrt{\frac{{k}_{B}T}{4\pi G{(\kappa {m}_{N})}^{2}{n}_{0}}}\simeq \frac{7.7}{\kappa }\cdot {10}^{18}\sqrt{\frac{T}{{n}_{0}}}\,(cm)$$

The total mass ${M}_{star}$ of the star is proportional to its volume, and to the average mass density given according to Eq. (8) as $\kappa {m}_{N}\langle {n}_{e}\rangle$ where $\langle {n}_{e}\rangle$ is the average electron density given approximately by $\approx \frac{{n}_{0}}{2}$. Then we get:

19 $${M}_{star}(star\,mass)\simeq \frac{4\pi }{3}{R}^{3}\kappa {m}_{N}\frac{{n}_{0}}{2}=1.6\cdot {10}^{33}\frac{{(T/(1Kelvin))}^{3/2}}{{\kappa }^{2}\sqrt{{n}_{0}}}\,(g)$$

Here we substituted the value of the star radius according to Eq. (18) and inserted an averaged value for the electron density $\langle {n}_{e}\rangle$ which is proportional to its density ${n}_{o}$ in the star center. In Eq. (18) and (19) we can plug in CGS unit for *T* and ${n}_{0}$, and the results for the radius and mass of the star are obtained, respectively. We find that the star mass is increasing with temperature as it is proportional to ${(T/(1Kelvin))}^{3/2}$. We find also the result that the star mass is inversely proportional to the square root of the density of the electrons in its center due to increase in its volume.

### Alternative derivation for the star radius

We give here an alternative derivation for the star radius in order to support our results using a different approximation. We start from Eq. (11), where we substitute, in this equation the following definitions:

20 $$y=\sqrt{4\pi G\kappa {m}_{N}{n}_{0}}\,r;\,z=\frac{\kappa {m}_{N}}{{k}_{B}T}$$

and express the potential function as a series expansion. Then this equation can be written as:

21 $$\frac{{d}^{2}\phi (y)}{d{y}^{2}}+\frac{2}{y}\frac{d\phi }{dy}=\{1-z\phi (y)+\frac{{z}^{2}\phi {(y)}^{2}}{2}-\frac{{z}^{3}\phi {(y)}^{3}}{3!}+\cdots \}.$$

The solution for $\phi (y)$ can be estimated by using the following series expansion:

22 $$\phi (y)=0+ay+b{y}^{2}+c{y}^{3}+d{y}^{4}\cdots $$

Inserting Eq. (22) into Eq. (21), and performing the derivatives of $\phi (y)$ we get:

23 $$(\frac{2a}{y}+6b+12cy+20d{y}^{2}+\cdots )=[1-z(ay+b{y}^{2}+\cdots )+{\frac{{z}^{2}{a}^{2}y}{2}}^{2}+\ldots ]$$

We obtain the coefficients $a,b,c,d,\cdots$ by equating the sum of terms with the same exponent of *y*. We get:

24 $$a=c=0;\,b=1/6;\,d=-\,\frac{1}{120}z,\cdots .$$

Since *z* is a small number we get for the solution of $\phi (y)$:

25 $$\phi (y)\simeq (1/6){y}^{2}=\frac{2}{3}\pi G\kappa {m}_{N}{n}_{0}{r}^{2}=\phi (r)$$

Substituting $\phi (y)$ of Eq. (25) into Eq. (9) for ${n}_{e}(r)$ we get:

26 $${n}_{e}(r)={n}_{0}\,\exp \,[-\frac{2}{3}\frac{\pi G{(\kappa {m}_{N})}^{2}{n}_{0}{r}^{2}}{{k}_{B}T}]$$

We get that under the condition ${r}^{2}=\frac{3}{2}\frac{{k}_{B}T}{\pi G{(\kappa {m}_{N})}^{2}{n}_{0}}={R}^{2}$ the density of the star ${n}_{e}(r)$ is reduced to $\frac{1}{e}{n}_{0}$ so that the radius *R* of this star can be estimated as

27 $$R=\sqrt{\frac{3}{2}\frac{{k}_{B}T}{\pi G{(\kappa {m}_{N})}^{2}{n}_{0}}}$$

So this estimation for the star radius is larger by factor $\sqrt{6}\simeq 2.45$ relative to the estimation made in Eq. (18) but both estimations lead to the same orders of magnitudes.

### Optical properties of low density stellar plasma related to thermal bremsstrahlung

Radiative processes in low density plasma at high temperatures are obtained mainly by bremsstrahlung effects representing the acceleration of a charge in the Coulomb field of another charge (usually the acceleration of electrons by the nuclear charge). In the present work we are interested in thermal bremsstrahlung: that is we average the single speed expressions over a thermal distribution of speeds. A full analysis of this process requires a quantum treatment but in some regimes we can use a classical analysis and the quantum effect result as corrections (Gaunt factors^11^) to the classical formulas. Following such approach the total power per unit volume emitted by spontaneous thermal emission integrated over frequencies is given in CGS units as^7^

28 $$\frac{dW}{dtdV}(erg\cdot {\text{sec}}^{-1}/c{m}^{3})={(\frac{2\pi {k}_{B}T}{3m})}^{1/2}\frac{{2}^{5}\pi {e}^{6}}{3hm{c}^{3}}{Z}^{2}{n}_{e}{n}_{i}{\bar{g}}_{B}\simeq 1.4\cdot {10}^{-27}\sqrt{T}\,{n}_{e}{n}_{i}{Z}^{2}{\bar{g}}_{B}$$

here ${n}_{e}(r)$ and ${n}_{i}(r)$ are the electron and ion density ($c{m}^{-3}$), respectively, which are function of the distance *r* from the star center. The quantum correction factor ${\bar{g}}_{B}$ is a frequency average of the Gaunt factor ${g}_{B}$ where it value is estimated approximately by 1.2^7^.

Thermal free-free bremsstrahlung power absorption per unit frequency *f* and unit volume, is given as^7^

29 $$\frac{dW}{dtdVdf}(erg/c{m}^{3})=4\pi {\alpha }_{f}{B}_{f}(T),$$

where ${\alpha }_{f}$ is the free-free absorption coefficient and ${B}_{f}(T)$ is the mean energy density at temperature T given by Planck law per unit frequency

30 $${B}_{f}(T)=\frac{2h{f}^{3}/{c}^{2}}{\exp (hf/{k}_{B}T)-1}.$$

The absorption coefficient is evaluated as

31 $${\alpha }_{f}=\frac{4{e}^{6}}{3mhc}{(\frac{2\pi }{3km})}^{1/2}{T}^{-1/2}{Z}^{2}{n}_{e}{n}_{i}{f}^{-3}(1-{e}^{-hf/{k}_{B}T}){\bar{g}}_{f}.$$

For $hf/{k}_{B}T\ll 1$ we are in Rayleigh regime for which $(1-{e}^{-hf/{k}_{B}T})\approx \frac{hf}{{k}_{B}T}$ and under this condition we get numerically^7^

32 $${\alpha }_{f}=0.018\cdot {T}^{-3/2}{Z}^{2}{n}_{e}{n}_{i}{f}^{-2}{\bar{g}}_{f},$$

where ${\bar{g}}_{f}$ is a small number under the condition $\frac{z{e}^{2}}{\hslash v}\le 1$ We notice that both Eqs. (28) and (32) are proportional to the product ${n}_{e}{n}_{i}$ so that for very low density plasmas the bremsstrahlung effects are very small. On the other hand by following the stability analysis made in the previous section we find that such low density stars have extremely large radius so that gravitational effects are significant. Although we made the stability analysis for a star with spherical symmetry we predict that low density plasmas with different structures will have also very large volumes.

## Summary and Discussion

The present results can be summarized as follows: In low density plasmas the balance between the ideal gas radiation pressure and gravitational forces leads to a large star radius so that although the mass density is quite small the star volume is extremely large leading to a large star mass with strong gravitational forces. We showed also that the absorption and/or emission effects for such plasms can be treated by theories about thermal Bremsstrahlung. As these effects are proportional to the product of the density of electrons ${n}_{e}$ times the density of ionized ions ${n}_{i}$ the plasma becomes transparent for very low densities. It seems that the present analysis describes a new kind of dark matter. This idea raises fundamental questions and in the present discussion I give answers to them:

- In astronomy, luminosity is defined as the total amount of electromagnetic energy emitted per unit time by a star, galaxy, or other astronomical object. One can use Hertzsprung–Russel diagram (HR diagram) for plotting the temperature of stars against their luminosity (see e.g.^20^ and references included). The question arises what is the relation between such diagrams and the present analysis? Our answer is that the HR diagram is related to Stephan-Boltzmann law giving the total energy being emitted per unit time and per unit area at all wavelengths of black body radiation given as $\sigma {T}^{4}$ where $\sigma$ is known as the Stephan-Boltzmann constant and T is the temperature (in Kelvins). For getting the illumination one needs to multiply the electromagnetic energy emitted by Stephan-Boltzmann law by an effective radiation emitting area. This method is applied to an idealized object which is perfectly opaque and non-reflecting and for dense plasmas such assumption is approximately correct. For low density plasmas the emission of radiation depends on the emissivity which is the ratio between the radiation emitted by a certain body and that of black body radiation. For electromagnetic energy incident on plasma conservation of energy implies the relation: Incident energy = Absorption energy + Transmitted energy + Reflected energy. For any material which is in thermal equilibrium assuming that Boltzmann statistics \is not disturbed by the incident radiation then the emissivity of the plasma should be equal to its absorptivity. For low density plasma (or any other material) in which the absorption vanishes the emissivity tends also to zero so that Stephan-Boltzmann law and consequently HR diagram does not apply for such case.
- Non-luminous matter which introduces strong gravitational waves (known as ‘dark matter’) was inferred already in 1937 by Swiss astronomer Fritz Zwicky^21^. The existence of this matter was confirmed later by Rubin, Thonnard & Ford^22^. Evidence for the existence of dark matter first came from the fact that luminous matter cannot account for velocities observed in rotation curves implying a significant invisible component. This fact can be related to the present low density plasma in the following way: Considering a small mass density $\rho$ rotating in circular motion around a big spherical mass *M* with a radius R the centrifugal force per unit volume is given by $\rho {v}_{rot}^{2}/R$ where ${v}_{rot}$ is the rotational velocity. For extremely large distance *R* this force decays due to the 1/*R* dependence but the ideal low density plasma pressure given by Eq. (1) remains to be approximately constant over extremely large radial distances. For very large distance from the star center the ideal gas pressure becomes the dominant repulsive force. In conclusion we expect the luminous matter to be embedded in low density plasma which extends over extremely large volumes. There is a strong similarity between the present work and Dark Matter Halos^23^. A dark Halo is the inferred Halo of invisible material that permeates and surrounds galaxies. The difference between the present model and that of dark Halos includes a difference in the dependence on physical parameters (In addition to difference in the physical models). For dark Halos the dependence on physical parameters was obtained by empirical equations in which the density of dark Halos was found to vary as power law in radius^24^. For low density plasma the density remains approximately constant up to a large distance *R* given approximately by Eq. (18) and beyond such radius it has an exponential decay. The exact way in which it decays has only a secondary effect on the low density plasma physical parameters which depends mainly on the star radius.

In the preset work I discussed the properties of low density plasmas by using approximate analytical equations. I hope in a future work to study numerical solutions to the fundamental Eq. (11) and make comparisons with experimental observations (e.g., those of gravitational lensing^25^) and with those obtained for dark Halos. Also I hope, in future work, to relate the present model to others cosmological models.
