# Author Correction: Transparency and stability of low density stellar plasma related to Boltzmann statistics and to thermal bremsstrahlung

**DOI:** 10.1038/s41598-020-64340-6

**Published:** 2020-04-24

**Authors:** Y. Ben-Aryeh

**Affiliations:** Technion-Israel Institute of Technology, Physic Department, Haifa, 32000 Israel

Correction to: *Scientific Reports* 10.1038/s41598-019-56784-2, published online 31 December 2019

The Acknowledgements section in this Article was omitted. The Acknowledgements section should read:

“The author would like to thank the anonymous Referees for their comments and advice on my previous article published in Scientific Reports 9, 20384 (2019). This helped me to develop the present paper. I would like also to thank Steve Lipson and Amos Ori for interesting discussions”

